# Radix Entomolaris in the Mandibular Molar Teeth of an Iranian Population

**DOI:** 10.1155/2017/9364963

**Published:** 2017-03-21

**Authors:** Maryam Kuzekanani, Laurence J. Walsh, Jahangir Haghani, Ali Zeynali Kermani

**Affiliations:** ^1^Endodontology Research Center, School of Dentistry, Kerman University of Medical Sciences & Health Services, Kerman, Iran; ^2^UQ Oral Health Centre, School of Dentistry, University of Queensland, Herston, QLD, Australia

## Abstract

*Purpose*. Supernumerary roots in permanent mandibular molar teeth make endodontic treatment more complicated. The aim of this study was to determine the prevalence of Radix Entomolaris (RE) in permanent mandibular first and second molars in the population of Kerman, in the southeast of Iran.* Materials and Methods*. From a collection of 500 mandibular first and second molar teeth extracted over 2015-2016 at dental clinics in Kerman, teeth were scored for an additional distolingual root, and the average root length and root morphology of this extra root were determined using the De Moor classification scheme.* Results*. In this population, RE occurred in 6% of mandibular first molars (4% with a straight apex (Type I) and 2% with buccal apical curvature (Type III)). In all cases, RE was the shortest root, with an average root length of 18.37 mm. RE occurred in only 0.8% of mandibular second molars, with an average root length of 18.0 mm. All mandibular second molars with RE were of Type III. Fisher's exact test showed that the difference in frequency between first and second molars was statistically significant (two-sided *P* = 0.002).* Conclusion*. Radix Entomolaris occurs more frequently in mandibular first molars than in mandibular second molars in this sample of 500 mandibular molars. The reported rate of 6% in first molars is expected to be higher than reported rates in European or Caucasian populations where the prevalence is typically less than 2%.

## 1. Introduction

A three-dimensional appreciation of root morphology and the associated root canal anatomy and the range of normal variations is essential for molar endodontic treatment [1]. The external root anatomy of teeth can vary, as well as the number of canals within each root [2–6]. Variations in mandibular molars away from the normal two roots are of particular interest, including additional roots on the distolingual aspect (termed Radix Entomolaris (RE)) or on the mesiobuccal side (Radix Paramolaris (RP)) [5, 6]. The prevalence of anatomical variations in teeth is influenced by ethnicity rather than by gender [7, 8].

Radiographic identification of additional roots present on mandibular molars is essential so that all root canals are identified and instrumented during endodontic treatment, since overlooked canals which have not undergone chemomechanical preparation will contribute to early failure of endodontic treatment. Use of cone beam volumetric tomography can be highly informative when anomalies in root morphology are identified on periapical radiographs. In the absence of such imaging, tube shifting with additional views taken from a more medial or distal angle of 20–30 degrees (Parallax technique) can help delineate the root morphology according to Buccal Object Rule [9–11].

Past studies of RE or RP have been case reports using periapical radiographs or small case series. Such work reveals a prevalence of RE of only 1 to 4 percent in European, African, and Caucasian populations, but as high as 30% in Mongolian or Asian populations [12–14].

**Figure 1:**
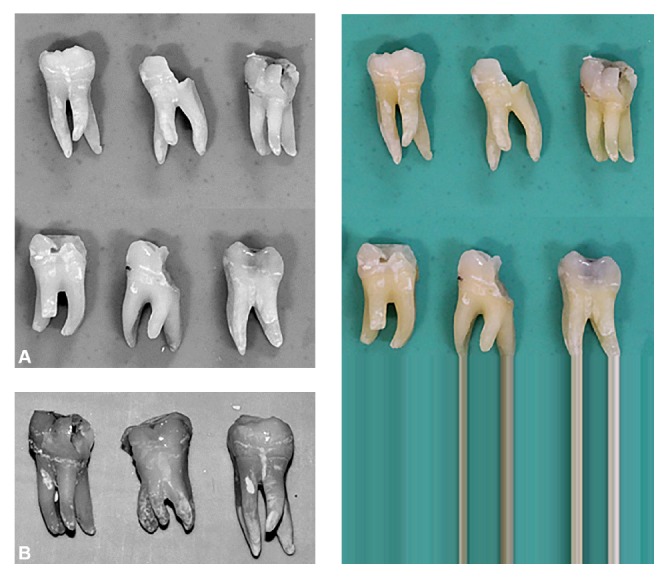
(A) Typical examples of mandibular molar teeth showing Radix Entomolaris. (A) First molars. (B) Second molars.

In mandibular molar teeth, the supernumerary roots of RE or RP can range from a short conical root to a root of normal length (Figure 1) [8]. From radiographic examination, three types of patterns for curvature of the supernumerary roots in RE have been described in the De Moor classification scheme: Type I (straight root), Type II (curved in the coronal third and then straight continuing to the apex), and Type III (curved in the coronal third, with mesiobuccal curvature from the middle third to the apical third of the root) [7, 8].

Given the known ethnic variations in supernumerary roots and the lack of data on contemporary populations in the Middle East, the present study followed a cross-sectional design to assess the occurrence of RE in the Iranian population, based on macroscopic examination of 500 extracted mandibular molar teeth, distributed equally between first and second molars.

## 2. Materials and Methods

With the approval of the institutional ethics committee (approval number K.93.421), extracted permanent mandibular molar teeth were collected in 2015–2106 from dental clinics in Kerman, Iran. All teeth had been removed because of complications of dental caries. No data was collected on gender or age of subjects having extractions. After excluding teeth with broken roots or roots which had been surgically removed, a sample of 250 sequential first and second molar teeth were collected. The teeth were disinfected with 2.5% sodium hypochlorite solution and then kept in distilled water until examined.

All teeth were examined macroscopically for supernumerary roots. When RE was identified as an additional root on the distolingual aspect of the mesial root, the type of RE was determined according to the De Moor classification and the root length measured (from the apex to the cusp tips) [8, 15], recognising that in cases of marked root curvature this could underestimate root length by 1 mm [16].

## 3. Results

Supernumerary roots in the Radix Entomolaris pattern were found in 15/250 (6%) of mandibular first molar teeth, and in 2/250 (0.8%) of mandibular second molar teeth. Using Fisher's exact test, the difference in frequency between first and second molars was statistically significant (two-sided *P* = 0.002).

The RE cases in first molars were divided into 10 cases (4%) with a straight apex (Type I) and 5 cases (2%) with buccal apical curvature (Type III). Both RE cases in second molars were Type III.

In terms of root length, in all teeth with RE, the supernumerary root was the shortest root of the tooth. In first molars, the average root length was 18.37 mm, while in second molars it was 18.0 mm.

## 4. Discussion

RE is not a common finding other than in Asian/Mongolian populations, where up to 30% of mandibular molar teeth can show additional roots. In the present cohort of Iranians from Kerman, which is in the southeast of the country, the rate of 6% in first molars is higher than reported rates in European or Caucasian populations where the prevalence is typically less than 2% [7, 8, 15–17].

A retrospective study performed on the full mouth radiographs of 500 patients of south Indian origin, reported an overall occurrence of 13.3% for Radix Entomolaris in this Asian population, which is still much lower than that of mongoloid origin. This study showed no significant difference between gender or side of occurrence but the bilateral prevalence of symmetric distribution of the RE in this South Asian group was 43.1% [18]. Moreover, the incidence of the RE, reported by L. A. Bahammam and H. A. Bahammam in a Saudi Arabian population which resulted from a study on a total of 280 extracted mandibular first molars, was 6.07%, almost the same as our rate in this southeast Iranian population. These very similar results between 2 near parts of the world again confirm the role of ethnicity in anatomic and morphologic variations of the teeth [19].

Another clinical study done by Mukhaimer and Azizi on a total of three hundred and twenty-two mandibular first molars scheduled for root canal treatment at the Dental Center of the Arab American university reported an overall incidence of 3.73% for Radix Entomolaris in a Palestinian group of patients which is within the range of previous reports from the Middle East but again significantly lower than the far east [20].

Shemesh et al. also in a retrospective study on a total of 1020 Israeli patients Cone Beam Computed Tomographic scans reported an overall incidence of 2.03% and 0.41 for Radix Entomolaris in mandibular first and second molars of this population, respectively. In 26% of the cases found, the RE happened bilaterally while there was no significant difference in the incidence of the RE between 2 sexes or side of occurrence [21].

Another retrospective study done on digital radiographs of 640 patients of a Turkish origin showed an overall incidence of 1.41% for Radix Entomolaris in mandibular first molars of this population with almost equal distribution between men and women. This rate of incidence is similar to that of Caucasian trait rather than the Asian [22]. Turkey is located in the northwest of Iran and just in far opposite point of Kerman. There is no evident report about the rate of Radix Entomolaris in mandibular molar teeth of north parts of Iran where the racial origins of the populations living there is rather similar to that of Turkey.

Clinically, signs of RE may include altered coronal anatomy, such as a more prominent distolingual lobe accompanied by a cervical convexity [7]. Nevertheless, it is important that sufficient radiographs are taken of mandibular molar teeth prior to either endodontics or extraction so that this important variation is not missed. Using preoperative periapical radiographs in a tube shift technique should assist clinicians in determining whether supernumerary roots are present on the lingual (RE) or buccal (RP) aspects, when cone beam volumetric tomography is not available to assist with making a diagnosis.

In mandibular molar teeth with RE, achieving successful root canal treatment requires that the traditional triangular outline of the access cavity be modified to a trapezoidal form with an extension towards the distolingual direction so as to gain straight line access into the canal of the additional root, for instrumentation and obturation [10]. Based on the present results, the root lengths at around 18 mm on average are some 3 mm shorter than the normal 21 mm distance from cusps to apex which would be expected; however this is less important clinically than the marked curvature which can occur in some cases [16, 17, 22, 23].

## 5. Conclusion

Radix Entomolaris occurs more frequently in mandibular first molars than in mandibular second molars in this population. The rate of 6% in first molars is higher than reported rates in European or Caucasian populations where the prevalence is typically less than 2% but much less than the mongoloid trait.
